# Circular RNA RHOT1 promotes progression and inhibits ferroptosis via mir-106a-5p/STAT3 axis in breast cancer

**DOI:** 10.18632/aging.202608

**Published:** 2021-03-03

**Authors:** Huiming Zhang, Zhicheng Ge, Zihan Wang, Yinguang Gao, Yang Wang, Xiang Qu

**Affiliations:** 1 Department of General Surgery, Beijing Friendship Hospital, Capital Medical University, Beijing, China

**Keywords:** breast cancer, progression, ferroptosis, circRHOT1, miR-106a-5p

## Abstract

To explore the effect of circRHOT1 on breast cancer progression and the underlying mechanism. Significantly, our data revealed that the depletion of circRHOT1 was able to repress the proliferation and induce the apoptosis of breast cancer cells. CircRHOT1 knockdown could remarkably inhibit the invasion and migration in the breast cancer cells. Meanwhile, the depletion of circRHOT1 enhanced the erastin-induced inhibition effect on cell growth of breast cancer cells. The circRHOT1 knockdown notably increased the levels of reactive oxygen species (ROS), iron, and Fe^2+^ in breast cancer cells. Mechanically, circRHOT1 was able to sponge microRNA-106a-5p (miR-106a-5p) and inhibited ferroptosis by down-regulating miR-106a-5p in breast cancer cells. Besides, miR-106a-5p induced ferroptosis by targeting signal transducer and activator of transcription 3 (STAT3) in the system. Moreover, the overexpression of STAT3 and miR-106a-5p inhibitor could reverse circRHOT1 knockdown-mediated breast cancer progression. Functionally, circRHOT1 promoted the tumor growth of breast cancer *in vivo*. In conclusion, we discovered that circRHOT1 contributed to malignant progression and attenuated ferroptosis in breast cancer by the miR-106a-5p/STAT3 axis. Our finding provides new insights into the mechanism by which circRHOT1 promotes the development of breast cancer. CircRHOT1 and miR-106a-5p may serve as potential targets for breast cancer therapy.

## INTRODUCTION

Breast cancer is the most frequent lethal cancer in women globally [1]. Despite the advancement of various treatments, breast cancer is still the second principal reason for tumor-related death in women [2]. Adjuvant therapy, radical surgery, and early diagnosis improve patients' survival times and prognosis with breast cancer, but mortality incidence is still unsatisfactory [3]. To develop the practical therapeutic strategy, the comprehensive understanding of the molecular signalings in breast cancer pathogenesis is urgently required [4]. Ferroptosis serves as a distinctive type of Regulated cell death (RCD) and is identified initially when a small molecule called erastin restrains the RCD process, especially in RAS-mutated cancer cells [5]. It has been identified that the inhibition of ferroptosis can enhance cancer progression, and ferroptosis plays a crucial role in breast cancer development [6–8]. However, the molecular mechanism of the ferroptosis process in breast cancer progression remains elusive.

With the rapid development of next-generation sequencing technology, circular RNAs (circRNAs), as a crucial sort of regulatory RNAs that forms a loop structure without 5′-3′ polyadenylated or polarities tails, have been identified [9]. CircRNAs demonstrate essential role in the modulation of breast cancer progression in various investigations [10, 11]. For example, it has been reported that circABCB10 contributes to progression and proliferation of breast cancer cells by targeting miR-1271 [12]. Circ001783 is able to modulate the malignant development of breast cancer through regulating miR-200c-3p [13]. Moreover, circRHOT1 has been reported to promote cancer progression in multiple cancer models, such as liver cancer and pancreatic cancer [14, 15]. It has been identified that circRHOT1 is abnormally expressed in the and promotes the proliferation, migration, and invasion, and inhibits apoptosis of cancer cells [15]. However, the role of circRHOT1 in breast cancer development remains unreported. Accordingly, we were interested in the effect of circRHOT1 on breast cancer.

MicroRNAs (miRNAs) have been well-recognized to regulate gene expression in various biological processes, such as invasion, migration, apoptosis, and proliferation [16]. Multiple investigations have demonstrated miRNAs are involved in the regulation of breast cancer. For instance, it has been reported that miR-183-5p reduces apoptosis and contributes to proliferation of human breast cancer by regulating the PDCD4 [17]. MiR-802 inhibits the proliferation of breast cancer by targeting FoxM1 [18]. Meanwhile, a recent study shows that miR-106a-5p as a tumor suppressor in breast cancer progression [19]. Moreover, STAT3 is a well-identified oncogenic regulator in breast cancer [20]. We identified the potential interaction of circRHOT1 with miR-106a-5p, miR-106a-5p with STAT3 in the bioinformatic analysis. However, the correlation of circRHOT1 with miR-106a-5p and STAT3 in the development of breast cancer is still elusive.

In this study, we focused on the exploration of the role and the underlying mechanism of circRHOT1 in the development of NSCLC. We identified a novel function of circRHOT1 promoting malignant progression and inhibiting ferroptosis in breast cancer by regulating the miR-106a-5p/STAT3 axis.

## RESULTS

### CircRHOT1 promotes proliferation and inhibits apoptosis of breast cancer cells

**Figure 1:**
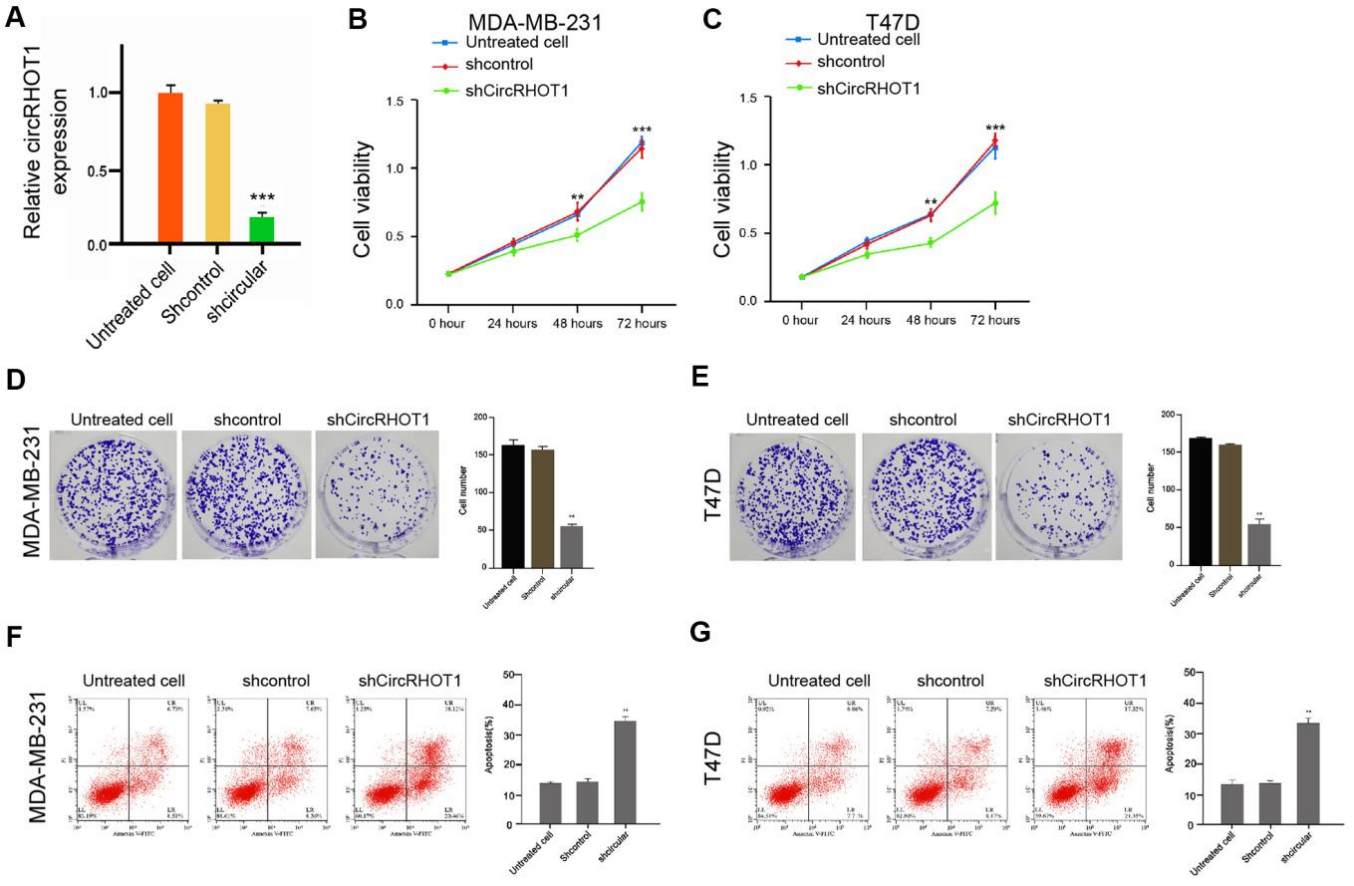
**CircRHOT1 promotes proliferation and inhibits apoptosis of breast cancer cells.** (**A**) The MDA-MB-231 cells were treated with control shRNA or circRHOT1 shRNA. The expression of circRHOT1 was measured by qPCR in the cells. (**B**–**G**) The MDA-MB-231 and T47D cells were treated with control shRNA or circRHOT1 shRNA. (**B**, **C**) The cell viability was measured by the MTT assays in the cells. (**D**, **E**) The cell proliferation was analyzed by the colony formation assays in the cells. (**F**, **G**) The cell apoptosis was assessed by flow cytometry analysis in the cells. Data are presented as mean ± SD. Statistic significant differences were indicated: ** *P* < 0.01.

To assess the potential function of circRHOT1 in the modulation of breast cancer progression, the human breast cancer MDA-MB-231 and T47D cells were treated with control shRNA or circRHOT1 shRNA, and the efficiency of circRHOT1 depletion was validated in the cells (Figure 1A). MTT assays revealed that the depletion of circRHOT1 inhibited the cell viability of the MDA-MB-231 and T47D cells (Figure 1B, 1C). Similarly, the colony formation was reduced by the circRHOT1 knockdown in the MDA-MB-231 and T47D cells (Figure 1D, 1E). Furthermore, cell apoptosis was enhanced by the circRHOT1 depletion in the cells (Figure 1F, 1G). Taken, together, these data suggest that circRHOT1 promotes proliferation and inhibits apoptosis of breast cancer cells.

### CircRHOT1 enhances invasion and migration of breast cancer cells

**Figure 2:**
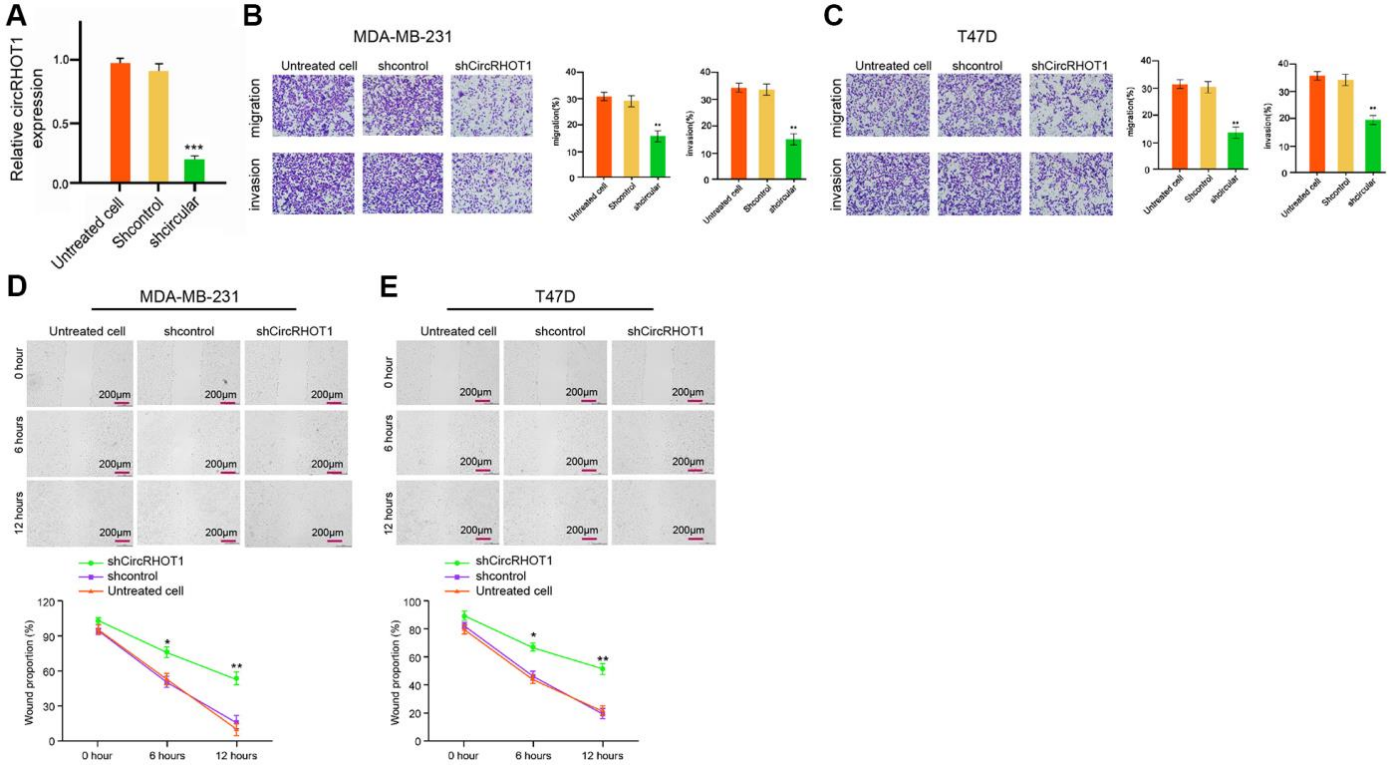
**CircRHOT1 enhances invasion and migration of breast cancer cells.** (**A**) The MDA-MB-231 cells were treated with control shRNA or circRHOT1 shRNA. The expression of circRHOT1 was measured by qPCR in the cells. (**B**–**D**) The MDA-MB-231 and T47D cells were treated with control shRNA or circRHOT1 shRNA. (**B**, **C**) The cell migration and invasion were analyzed by transwell assays in the cells. (**D**, **E**) The migration and invasion were examined by wound healing assays in the cells. The wound healing proportion was shown. Data are presented as mean ± SD. Statistic significant differences were indicated: ** *P* < 0.01.

Next, we investigated the effect of circRHOT1 on the migration and invasion of breast cancer cells. For this purpose, the MDA-MB-231 and T47D cells were treated with control shRNA or circRHOT1 shRNA, and the efficiency of circRHOT1 depletion was confirmed in the cells (Figure 2A). Transwell assays revealed that the migration and invasion of MDA-MB-231 and T47D cells were significantly repressed by the depletion of circRHOT1 (Figure 2B, 2C). Similarly, the depletion of circRHOT1 remarkably enhanced the wound healing proportion in the cells (Figure 2D, 2E), suggesting that circRHOT1 is able to enhance the migration and invasion of breast cancer cells.

### CircRHOT1 reduces ferroptosis in breast cancer cells

**Figure 3:**
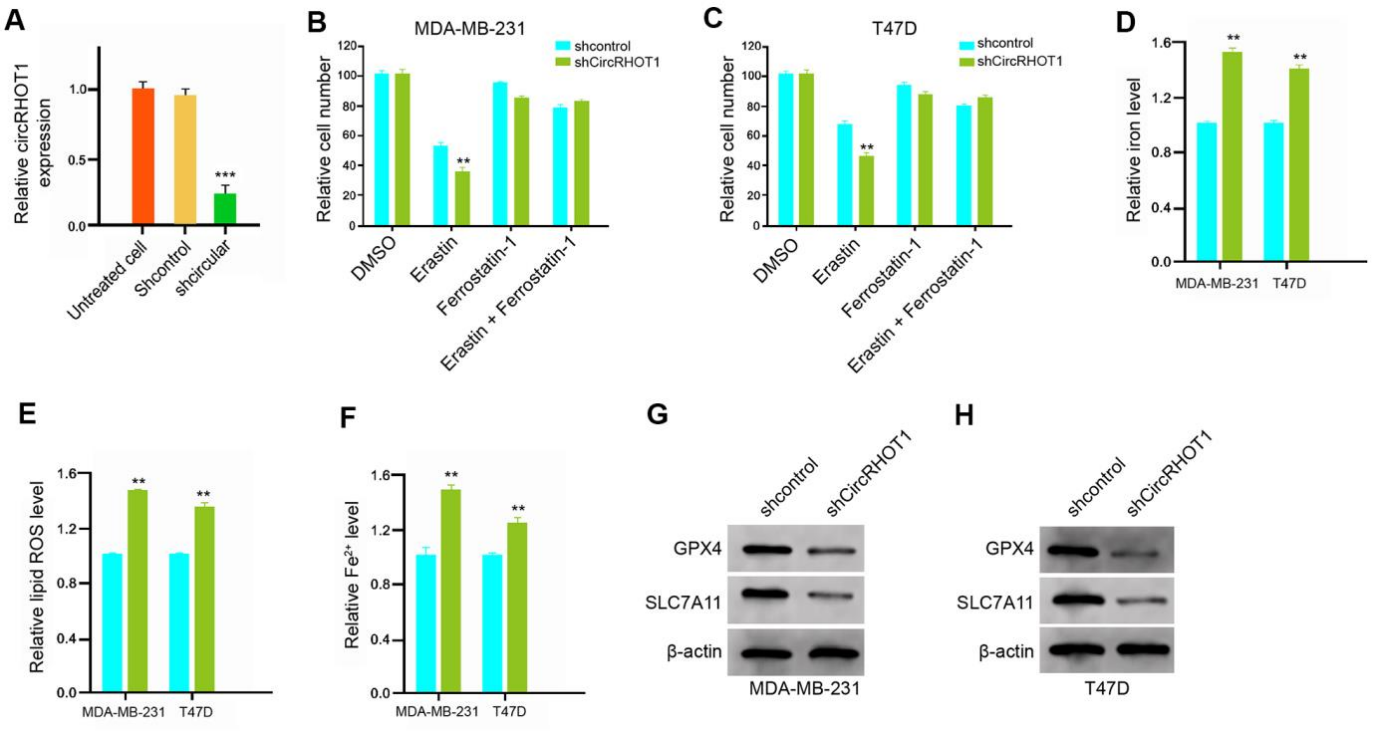
**CircRHOT1 reduces ferroptosis in breast cancer cells.** (**A**) The MDA-MB-231 cells were treated with control shRNA or circRHOT1 shRNA. The expression of circRHOT1 was measured by qPCR in the cells. (**B**, **C**) The MDA-MB-231 and T47D were co-treated with 5 mmol/L erastin or ferrostatin (1 mmol/L) and circRHOT1 shRNA. The cell growth was measured by MTT assays. (**D**–**H**) The MDA-MB-231 and T47D cells were treated with control shRNA or circRHOT1 shRNA. (**D**) The levels of ROS were analyzed by flow cytometry analysis in the cells. (**E**, **F**) The levels of iron and Fe2+ were tested by Iron Assay Kit. (**G**, **H**) The expression of GPX4, SLC7A11, and β-actin was assessed by Western blot analysis in the cells. Data are presented as mean ± SD. Statistic significant differences were indicated: ** *P* < 0.01.

To assess the role of circRHOT1 in ferroptosis, we analyzed the effect of circRHOT1 on the erastin-induced inhibition of cell growth and the intracellular levels of reactive oxygen species, iron, and Fe^2+^, and the expression of GPX4 and SLC7A11, which was the markers for ferroptosis. To this end, the MDA-MB-231 and T47D cells were treated with control shRNA or circRHOT1 shRNA, and the efficiency of circRHOT1 knockdown was validated in the cells (Figure 3A). Significantly, the depletion of circRHOT1 reinforced the erastin-induced suppression of cell growth in the MDA-MB-231 and T47D cells, in which erastin served as the activator of ferroptosis (Figure 3B, 3C). Meanwhile, the iron levels were increased by circRHOT1 depletion in the cells (Figure 3D). The circRHOT1 knockdown remarkably enhanced the levels of ROS in the MDA-MB-231 and T47D cells (Figure 3E). Besides, the depletion of circRHOT1 promoted the accumulation of Fe^2+^ in the cells (Figure 3F). Moreover, the expression of GPX4 and SLC7A11 was attenuated by circRHOT1 knockdown in the cells (Figure 3G, 3H). Together these indicate that circRHOT1 can reduce ferroptosis in breast cancer cells.

### CircRHOT1 inhibits ferroptosis by sponging miR-106a-5p in breast cancer cells

**Figure 4:**
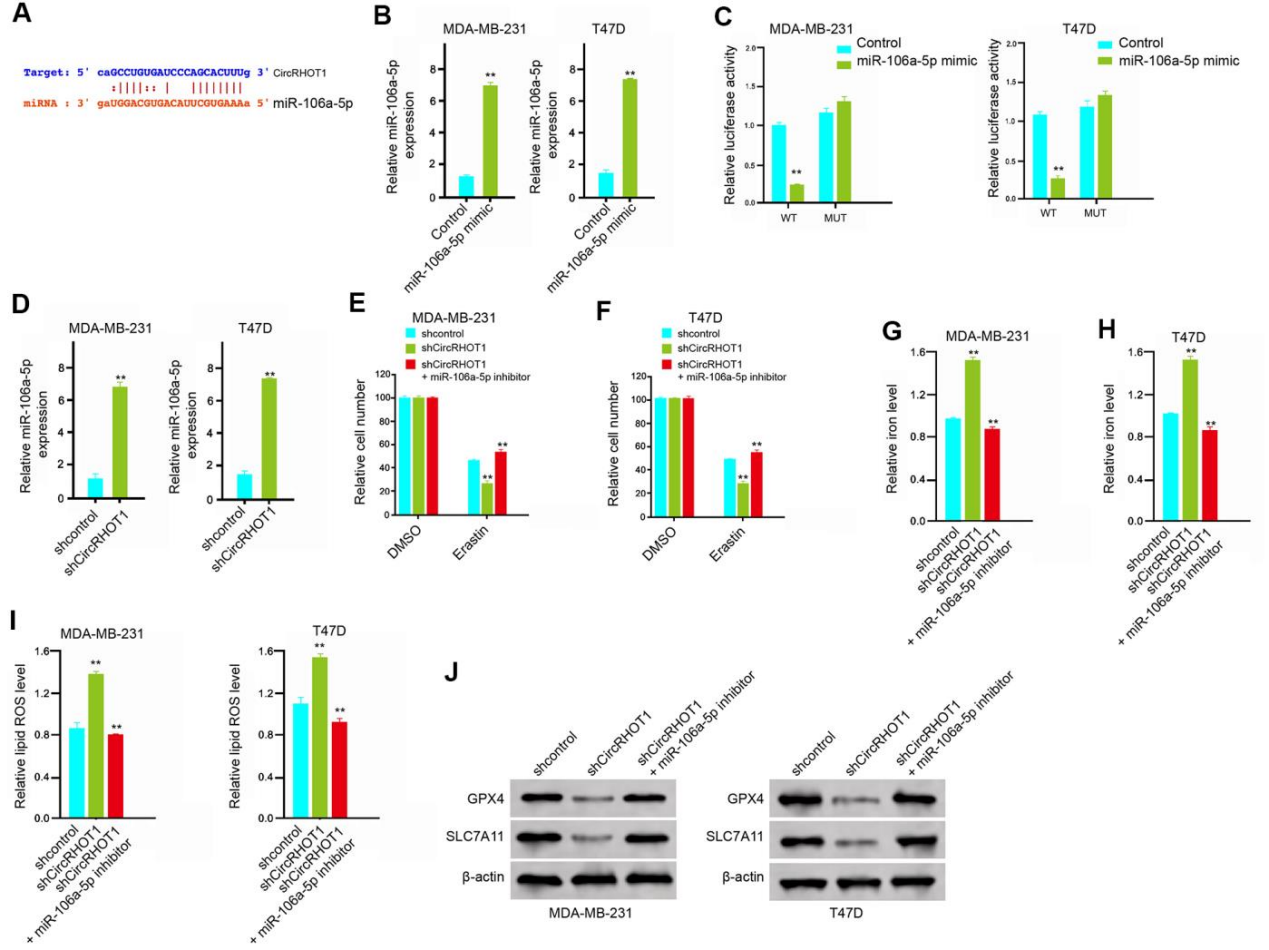
**CircRHOT1 inhibits ferroptosis by sponging miR-106a-5p in breast cancer cells.** (**A**) The potential interaction between circRHOT1 and miR-106a-5p was identified by the bioinformatic analysis using ENCORI (http://starbase.sysu.edu.cn/index.php). (**B**, **C**) The MDA-MB-231 and T47D cells were treated with the miR-106a-5p mimic or control mimic. (**B**) The expression levels of miR-106a-5p were measured by qPCR in the cells. (**C**) The luciferase activities of wild type circRHOT1 (circRHOT1 WT) and circRHOT1with the miR-106a-5p-binding site mutant (circRHOT1 MUT) were determined by luciferase reporter gene assays in the cells. (**D**) The MDA-MB-231 and T47D cells were treated with control shRNA or circRHOT1 shRNA. The expression of miR-106a-5p was analyzed by qPCR in the cells. (**E**, **F**) The MDA-MB-231 and T47D cells were treated with 5 mmol/L erastin, co-treated with 5 mmol/L erastin and circRHOT1 shRNA, or o-treated with 5 mmol/L erastin, circRHOT1 shRNA, and miR-106a-5p inhibitor. The cell growth was analyzed by MTT assays. (**G**–**J**) The MDA-MB-231 and T47D cells were treated control shRNA, circRHOT1 shRNA, or co-treated with circRHOT1 shRNA and miR-106a-5p inhibitor. (**G**, **H**) The levels of iron were analyzed by Iron Assay Kit. (**I**) The levels of ROS were measure by flow cytometry analysis in the cells. (**J**) The expression of GPX4, SLC7A11, and β-actin was measured by Western blot analysis in the cells. Data are presented as mean ± SD. Statistic significant differences were indicated: ** *P* < 0.01.

Then, we further explored the mechanism of circRHOT1-mediated breast cancer progression. We identified the potential interaction between circRHOT1 and miR-106a-5p in the bioinformatic analysis by using ENCORI (http://starbase.sysu.edu.cn/index.php) (Figure 4A). Then, we treated the MDA-MB-231 and T47D cells with miR-106a-5p mimic or the control mimic, and the efficiency was verified in the cells (Figure 4B). The miR-106a-5p mimic remarkably reduced the luciferase activities of circRHOT1 but failed to affect circRHOT1 with the miR-106a-5p-binding site mutant (Figure 4C). Meanwhile, the depletion of circRHOT1 significantly enhanced the expression of miR-106a-5p in the MDA-MB-231 and T47D cells (Figure 4D). Moreover, the miR-106a-5p inhibitor could rescue circRHOT1 depletion-inhibited cell growth in the erastin-treated MDA-MB-231 and T47D cells (Figure 4E, 4F). Similarly, miR-106a-5p inhibitor reversed circRHOT1 depletion-enhanced levels of iron and ROS in the system (Figure 4G–4I). In addition, the circRHOT1 knockdown-reduced expression of GPX4 and SLC7A11 was enhanced by miR-106a-5p inhibitor (Figure 4J). Taken together, these data results suggest that circRHOT1 inhibits ferroptosis by sponging miR-106a-5p in breast cancer cells.

### MiR-106a-5p induces ferroptosis by targeting STAT3 in breast cancer cells

**Figure 5:**
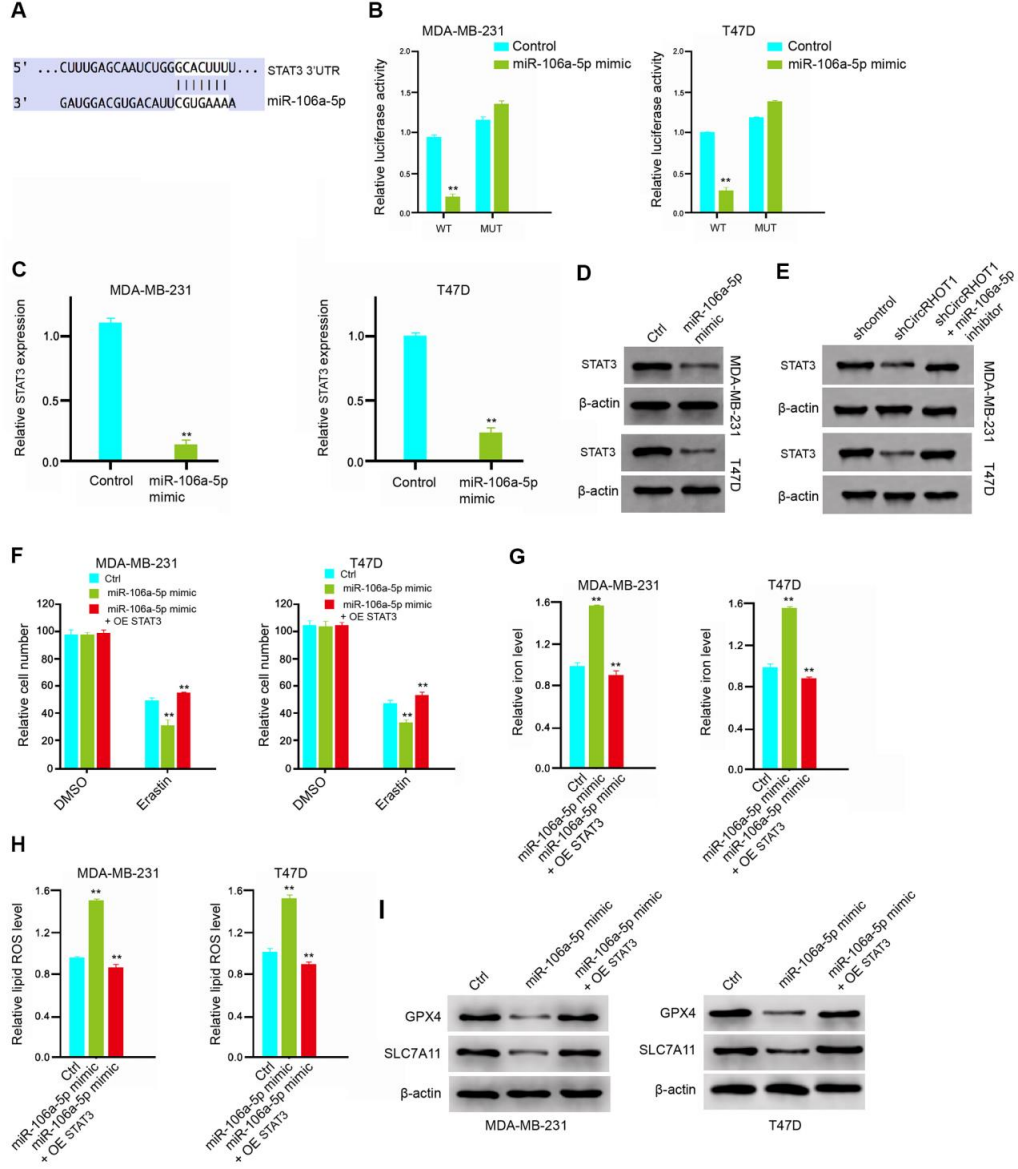
**MiR-106a-5p induces ferroptosis by targeting STAT3 in breast cancer cells.** (**A**) The interaction of miR-106a-5p and STAT3 3’ UTR was identified by bioinformatic analysis using Targetscan (http://www.targetscan.org/vert_72/). (**B**–**D**) The MDA-MB-231 and T47D cells were treated with the miR-106a-5p mimic or control mimic. (**B**) The luciferase activities of wild type STAT3 (STAT3 WT) and STAT3 with the miR-106a-5p-binding site mutant (STAT3 MUT) were determined by luciferase reporter gene assays in the cell. (**C**) The mRNA expression of STAT3 was analyzed by qPCR in the cells. (**D**) The protein expression of STAT3 and β-actin was tested by Western blot analysis in the cells. (**E**) The MDA-MB-231 and T47D cells were treated control shRNA, circRHOT1 shRNA, or co-treated with circRHOT1 shRNA and miR-106a-5p inhibitor. The protein expression of STAT3 and β-actin was assessed by Western blot analysis in the cells. (**E**, **F**) The MDA-MB-231 and T47D cells were treated with 5 mmol/L erastin, co-treated with 5 mmol/L erastin and miR-106a-5p mimic, or o-treated with 5 mmol/L erastin, miR-106a-5p mimic, and pcDNA.1-STAT3. The cell growth was analyzed by MTT assays. (**G**–**I**) The MDA-MB-231 and T47D cells were treated control shRNA, miR-106a-5p mimic, or co-treated with miR-106a-5p mimic and pcDNA.1-STAT3. (**G**) The levels of iron were analyzed by Iron Assay Kit. (**H**) The levels of ROS were measure by flow cytometry analysis in the cells. (**I**) The expression of GPX4, SLC7A11, and β-actin was measured by Western blot analysis in the cells. Data are presented as mean ± SD. Statistic significant differences were indicated: ** *P* < 0.01.

Then, we further explored the role of miR-106a-5p in the modulation of ferroptosis. We identified the miR-106a-5p-targeted site in STAT3 3’ UTR in a bioinformatic analysis by using Targetscan (http://www.targetscan.org/vert_72/) (Figure 5A). Significantly, the miR-106a-5p mimic treatment attenuated luciferase activities of wild type STAT3 but failed to affect the STAT3 with the miR-106a-5p-binding site mutant in the MDA-MB-231 and T47D cells (Figure 5B). Besides, the mRNA and protein expression of STAT3 were significantly repressed by miR-106a-5p mimic in the cells (Figure 5C, 5D), suggesting that miR-106a-5p is able to target STAT3 in the breast cancer cells. Meanwhile, the depletion of circRHOT1 significantly reduced the expression of STAT3, in which miR-106a-5p inhibitor could reverse this effect (Figure 5E). Furthermore, the overexpression of STAT3 could rescue miR-106a-5p mimic-inhibited cell growth in the erastin-treated MDA-MB-231 and T47D cells (Figure 5F). Similarly, STAT3 overexpression reversed miR-106a-5p mimic-enhanced levels of iron and ROS in the system (Figure 5G, 5H). In addition, the miR-106a-5p mimic-reduced expression of GPX4 and SLC7A11 was enhanced by the overexpression of STAT3 (Figure 5I). Together these data suggest that miR-106a-5p induces ferroptosis by targeting STAT3 in breast cancer cells.

### CircRHOT1 contributes to breast cancer progression by miR-106a-5p/STAT3 axis

**Figure 6:**
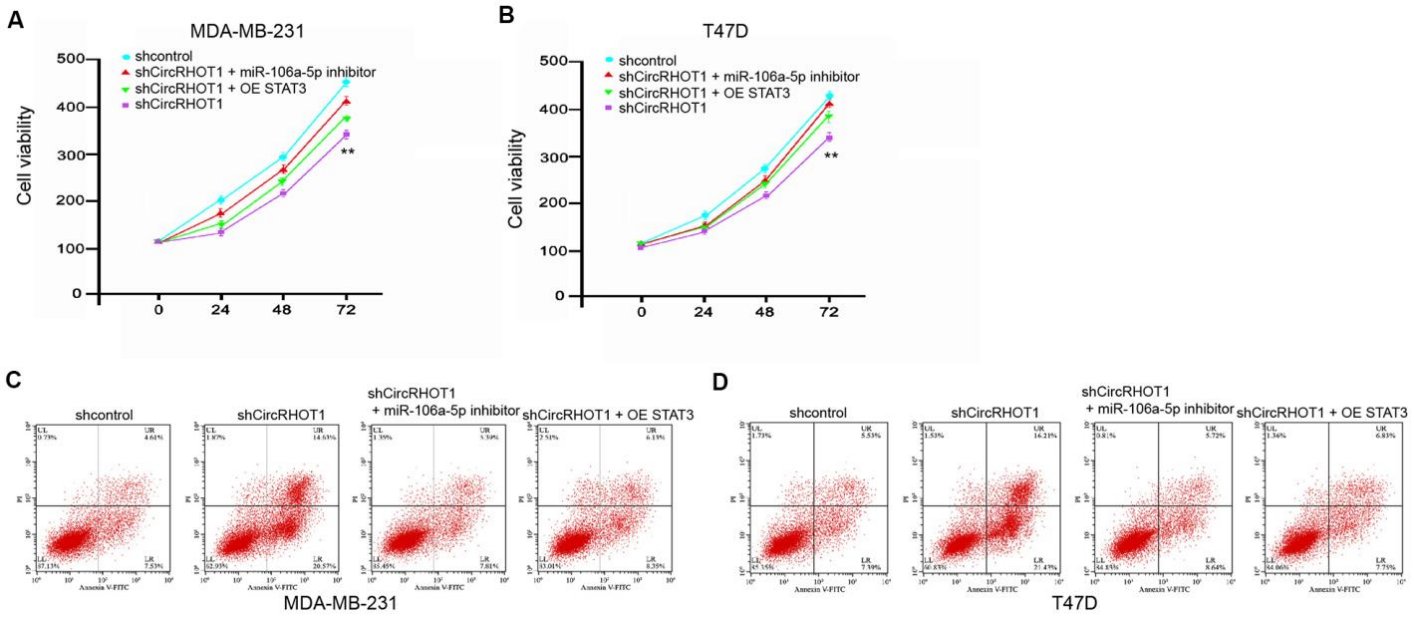
**CircRHOT1 contributes to breast cancer progression by miR-106a-5p/STAT3 axis.** (**A**–**D**) The MDA-MB-231 and T47D cells were treated control shRNA, circRHOT1 shRNA, or co-treated with circRHOT1 shRNA and miR-106a-5p inhibitor or pcDNA.1-STAT3. (**A**, **B**) The cell viability was measured by MTT assays in the cells. (**C**, **D**) The cell apoptosis was measure by flow cytometry analysis in the cells. Data are presented as mean ± SD. Statistic significant differences were indicated: ** *P* < 0.01.

Next, we further investigated the role of circRHOT1/miR-106a-5p/STAT3 axis in the modulation of breast cancer progression. As expected, the overexpression of STAT3 or miR-106a-5p inhibitor could enhance the circRHOT1 knockdown-reduced cell viability in the MDA-MB-231 and T47D cells (Figure 6A, 6B). Meanwhile, the cell apoptosis was enhanced by the depletion of circRHOT1, in which the overexpression of STAT3 or miR-106a-5p inhibitor could reverse this effect in the MDA-MB-231 and T47D cells (Figure 6C, 6D). Taken together, these data suggest that circRHOT1 contributes to breast cancer progression by miR-106a-5p/STAT3 axis.

### CircRHOT1 promotes the tumor growth of breast cancer *in vivo*

**Figure 7:**
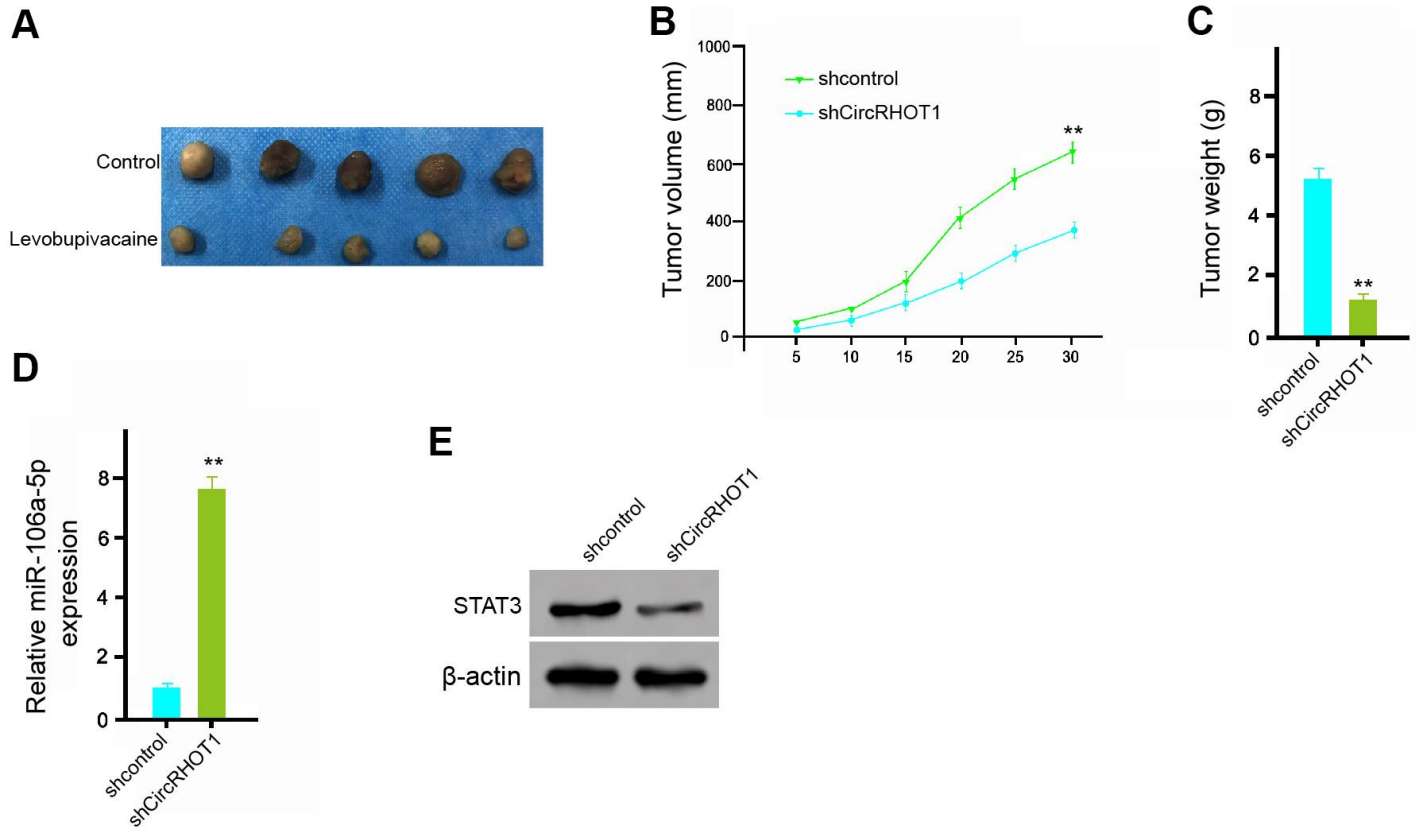
**CircRHOT1 promotes the tumor growth of breast cancer *in vivo*.** (**A**–**E**) The effect of circRHOT1 on tumor growth of breast cancer cells *in vivo* was analyzed by nude mice tumorigenicity assay by injected with the MDA-MB-231 cells treated with control shRNA or circRHOT1 shRNA. (**A**) Representative images of dissected tumors from nude mice were presented. (**B**) The average tumor volume was calculated and shown. (**C**) The average tumor weight was calculated and shown. (**D**) The expression levels of miR-106a-5p were measured by qPCR in the tumor tissues of the mice. (**E**) The protein expression of STAT3 and β-actin was assessed by Western blot analysis in the tumor tissues of the mice. N = 5. Data are presented as mean ± SD. Statistic significant differences were indicated: ** *P* < 0.01.

We further investigated the impact of circRHOT1 on the breast cancer development *in vivo*. For this purpose, we performed the tumorigenicity analysis in nude mice injected with MDA-MB-231 cells treated with control shRNA or circRHOT1 shRNA. The depletion of circRHOT1 significantly repressed the tumor growth of MDA-MB-231 cells *in vivo*, as demonstrated by the tumor size (Figure 7A), tumor volume (Figure 7B), and tumor weight (Figure 7C). Besides, the expression of miR-106a-5p was increased but the expression of STAT3 was decreased by the circRHOT1 depletion in the tumor tissues of the mice (Figure 7D, 7E). Together these indicate that circRHOT1 promotes the tumor growth of breast cancer *in vivo*.

## DISCUSSION

Breast cancer is the prevailing women cancer affecting modern people and resulting in high mortality [1]. CircRNAs have been identified to exert crucial functions in the modulation of breast cancer [12]. Nevertheless, the role of circRHOT1in the development of breast cancer is still unreported. In this study, we firstly identified that circRHOT1 could inhibit ferroptosis and promote the progression of breast cancer by modulating the miR-106a-5p/STAT3 axis.

Previous studies have identified several circRNAs in the regulation of breast cancer pathogenesis. It has been reported that circ100219 enhances the progression of breast cancer by sponging miR-485-3p [21]. CircMTO1 represses cell viability of and attenuates monastrol resistance by modulating the TRAF4/Eg5 signaling in breast cancer [22]. Circ0052112 increases invasion and migration of breast cancer cells by targeting miR-125a-5p [23]. Circ0001982 enhances the carcinogenesis of breast cancer by inhibiting miR-143 [24]. Circ0072309 suppresses invasion and proliferation by regulating miR-492 in breast cancer [25]. Moreover, it has been found that circRHOT1 contributes to the malignant progression of liver cancer by inhibiting NR2F6 expression [14]. The elevated circRHOT1 enhances invasion and proliferation of pancreatic cancer cells [15]. In this study, we demonstrated that circRHOT1 promoted proliferation and invasion, and migration, and inhibited apoptosis and ferroptosis in the breast cancer cells. CircRHOT1 enhanced the tumor growth of breast cancer *in vivo*. Our data elucidate a novel role of circRHOT1 in the breast cancer progression, providing crucial evidence of the function of circRNAs in modulating breast cancer. Our data are consistent with the previous reports that circRHOT1 is able to promotes proliferation and invasion, and migration, and inhibit apoptosis of cancer cells. Besides, we firstly identified that circRHOT1 can attenuate ferroptosis of breast cancer, presenting a novel function of circRHOT1 in cancer progression.

MiRNAs play crucial function in breast cancer progression. It has been reported that miR-135a enhances invasion and migration of breast cancer cells by regulating HOXA10 [26]. MiR-421 is able to target PDCD4 and regulate cell proliferation of breast cancer [27]. The inhibition of miR-141 regulates cell invasion and migration in breast cancer cells by targeting ANP32E [28]. MiR-218 represses invasion and migration of breast cancer cells by Slit2/Robo1 signaling [29]. Moreover, it has been reported that miR-106a-5p/SCN3A axis is involved in long noncoding RNA HOXA-AS2-regulated breast cancer [19]. Our mechanical investigation revealed that circRHOT1 repressed ferroptosis by sponging miR-106a-5p and miR-106a-5p induced ferroptosis by targeting STAT3 in the breast cancer cells. The overexpression of STAT3 and miR-106a-5p inhibitor reversed circRHOT1 depletion-inhibited proliferation and circRHOT1 depletion-enhanced apoptosis of breast cancer cells. These data uncover an unreported correlation of circRHOT1 with miR-106a-5p and STAT3 and provide new evidence that circRHOT1/miR-106a-5p/STAT3 signaling is critical for the modulation of breast cancer.

## CONCLUSIONS

In conclusion, we discovered that circRHOT1 contributed to malignant progression and attenuated ferroptosis in breast cancer by the miR-106a-5p/STAT3 axis. Our finding provides new insights into the mechanism by which circRHOT1 promotes the development of breast cancer. CircRHOT1 and miR-106a-5p may serve as potential targets for breast cancer therapy.

## MATERIALS AND METHODS

### Cell culture

The MDA-MB-231 and T47D cells were purchased in American Type Tissue Culture Collection. The cells were cultured in the medium of DMEM (Gibco, USA) with 0.1 mg/mL streptomycin (Gibco, USA), 100 units/mL penicillin (Gibco, USA), and 10% fetal bovine serum (Gibco, USA), under the condition of 37° C with 5% CO_2_. The lentiviral plasmids carrying circRHOT1 shRNA, the corresponding control shRNA, the pcDNA3.1-circRHOT1 overexpression vector, the pcDNA3.1-STAT3 overexpression vector, miR-106a-5p mimic, miR-106a-5p inhibitor, and corresponding control were synthesized and purchased (GenePharma, China) (GenScript, China). The transfection in the cells was performed by Liposome 3000 (Invitrogen, USA) according to the manufacturer's instructions.

### MTT assays

The cell proliferation was tested by MTT assays in the MDA-MB-231 and T47D cells. Sgotly, about 2×10^5^ cells were plated in 96-well plates and incubated for 24 hours. To assess the cell viability, the cells were cultured with the MTT solution (5 mg/mL) and incubated for 4 hours. And then 150 μL DMSO was applied to treat the cells. The cell viability was measured at 570nm absorbance by applying ELISA browser (Bio-Tek EL 800, USA).

### Colony formation assays

About 1×10^4^ MDA-MB-231 and T47D cells were put into 6-well plates and cultured in DMEM at 37° C. The cells were cleaned with PBS Buffer after 2 weeks, and made in methanol about thirty minutes, and dyed with crystal violet dye at the dose of 1%, after which the number of colonies was calculated.

### Transwell assays

Transwell assays was used to analyze the cell migration and invasion of MDA-MB-231 and T47D cells based on a Transwell plate (Corning, USA) according to the manufacturer's guidance. Shortly, the upper chambers were plated with around 1 × 10^5^ cells. Then solidified using paraformaldehyde (4%) and dyed using crystal violet. Invaded and migrated cells were recorded and calculated.

### Wound healing assay

About 3 × 10^5^ MDA-MB-231 and T47D cells were plated into the 24-well plates and incubated overnight to reach a full confluent as a monolayer. A 20μl pipette tip was applied to slowly cut a straight line across the well. Then the well was washed by PBS 3 times and changed with the serum-free medium and continued to culture. The wound healing percentage was calculated.

### Analysis of cell apoptosis

About 2×10^5^ MDA-MB-231 and T47D cells were plated in the 6-well dishes. Cell apoptosis was assessed by employing the Annexin V-FITC Apoptosis Detection Kit (CST, USA) using the manufacture’s instruction. Shortly, about 2×10^5^ collected and washed cells collected by binding buffer and were dyed at 25° C, followed by the flow cytometry analysis.

### Ferroptosis analysis

The cells were co-treated with 5 mmol/L erastin or ferrostatin (1 mmol/L). The cell viability was analyzed by MTT assays in the MDA-MB-231 and T47D cells. The reactive oxygen species production was measured by flow cytometry analysis in the cells. The levels of iron and Fe^2+^ were analyzed by Iron Assay Kit in the cells.

### Luciferase reporter gene assay

The luciferase reporter gene assays were performed by using the Dual-luciferase Reporter Assay System (Promega, USA). Briefly, the MDA-MB-231 and T47D cells were treated with the miR-106a-5p mimic, miR-106a-5p mimic or control mimic, pmirGLO-circRHOT1, pmirGLO-circRHOT1 mutant, pmirGLO-STAT3, and pmirGLO-STAT3 mutant were transfected in the cells by using Lipofectamine 3000 (Invitrogen, USA), followed by the analysis of luciferase activities, in which Renilla was applied as a normalized control.

### Quantitative reverse transcription-PCR (qRT-PCR)

The total RNAs were extracted by TRIZOL (Invitrogen, USA). The first-strand cDNA was manufactured as the manufacturer's instruction (Thermo, USA). The qRT-PCR was carried out by applying SYBR Real-time PCR I kit (Takara, Japan). The standard control for miRNA and mRNA/circRNA was U6 and GAPDH, respectively. Quantitative determination of the RNA levels was conducted in triplicate independent experiments. The primer sequences are as follows: circRHOT1 forward: 5′- ATCACCATTCCAGCTGATGT-3′, reverse: 5′- TGCTGTCTTTGTCTGTTCTTTC-3′; miR-106a-5p forward: 5′- GATGCTCAAAAAGTGCTTACAGTGCA -3′, reverse: 5′- TATGGTTGTTCTGCTCTCTGTCTC -3′; STAT3 forward: 5′- GGCCATCTTGAGCACTAAGC -3′, reverse: 5′-CGGACTGGATCTGGGTCTTA -3′; GAPDH forward: 5′-TATGATGATATCAAGAGGGTAGT-3′, reverse: 5′-TATGATGATATCAAGAGGGTAGT-3′; U6 forward: 5′-CTCGCTTCGGCAGCACA-3′, U6 reverse: 5′-AACGCTTCACGAATTTGCGT-3′.

### Western blot analysis

Total proteins were obtained from the mice tissues or cells with RIPA buffer (CST, USA). Protein concentrations were analyzed by applying the BCA Protein Quantification Kit (Abbkine, USA). Same concentration of protein was divided by SDS-PAGE (12% polyacrylamide gels), transferred to PVDF membranes (Millipore, USA) in the subsequent step. The membranes were hindered with 5% milk and hatched overnight at 4° C with the primary antibodies for STAT3 (Abcam, USA), GPX4 (Abcam, USA), SLC7A11 (Abcam, USA), and β-actin (Abcam, USA), in which β-actin served as the control. Then, the corresponding second antibodies (Abcam, USA) were used for hatching the membranes 1 hour at room temperature, followed by the visualization by using an Odyssey CLx Infrared Imaging System.

### Analysis of tumorigenicity in nude mice

The effect of levobupivacaine on tumor growth of breast cancer *in vivo* was assessed in the Balb/c nude mice (male, 4-week-old) (n=5). About 1×10^7^ cells MDA-MB-231 cells transfected with control shRNA or circRHOT1 shRNA were subcutaneously injected into the mice. After 5 days of injection, we measured tumor growth every 5 days. We sacrificed the mice after 30 days of injection, and tumors were scaled. Tumor volume was observed by estimating the length and width with calipers and measured with the method × 0.5. Animal care and method procedure were authorized by the Animal Ethics Committee of Beijing Friendship Hospital.

### Statistical analysis

Data were expressed as mean ± SD, and the statistical analysis was conducted using GraphPad prism 7. The unpaired Student’s *t*-test was used to compare two groups, and the one-way ANOVA was used to compare among multiple groups. *P* < 0.05 were considered as statistically significant.
